# HIF-1α Modulates Core Metabolism and Virus Replication in Primary Airway Epithelial Cells Infected with Respiratory Syncytial Virus

**DOI:** 10.3390/v12101088

**Published:** 2020-09-26

**Authors:** Dorothea R. Morris, Yue Qu, Anurodh Agrawal, Roberto P. Garofalo, Antonella Casola

**Affiliations:** 1 Department of Microbiology and Immunology, University of Texas Medical Branch, Galveston, TX 77555, USA; dormorri@utmb.edu (D.R.M.); rpgarofa@utmb.edu (R.P.G.); 2 Division of Clinical and Experimental Immunology and Infectious Disease (CEIID), Department of Pediatrics, University of Texas Medical Branch, Galveston, TX 77555, USA; yuqu@utmb.edu (Y.Q.); anagrawa@utmb.edu (A.A.)

**Keywords:** respiratory syncytial virus, RSV, metabolism, HIF-1α, HIF-2α

## Abstract

Metabolic reprogramming of host cells is key to the foundation of a successful viral infection. Hypoxia inducible factors (HIFs) mediate oxygen utilization by regulating cellular metabolism and redox homeostasis. Under normoxic conditions, HIF proteins are synthesized and subsequently degraded following ubiquitination to allow for normal metabolic activities. Recent studies suggest that respiratory syncytial virus (RSV) has the ability to induce HIF-1α stabilization and accumulation through non-hypoxic mechanisms. This makes the HIF pathway a potential avenue of approach for RSV therapeutic development. Using a model of primary human small alveolar epithelial cells, we demonstrate RSV infections to greatly alter cellular metabolism in favor of the glycolytic and pentose phosphate pathways. Additionally, we show RSV infections to stabilize HIF-1α and HIF-2α expression in these cells. Inhibition of HIF-1α, but not HIF-2α, was found to significantly reduce RSV replication as well as the glycolytic pathway, as measured by the expression of hexokinase II. Our study contributes to the understanding of RSV-mediated changes to cellular metabolism and supports further investigation into anti-HIF-1α therapeutics for RSV infections.

## 1. Introduction

Respiratory syncytial virus (RSV) is the etiological agent for almost one third of all acute lower respiratory infections globally [1]. Belonging to the family *Pneumoviridae*, RSV is a negative-sense single-stranded RNA virus that encodes 11 viral proteins [2,3]. The spread of RSV is accomplished through the inhalation of aerosolized droplets, allowing for inoculation of the nasopharyngeal mucosa. This is followed by the rapid descent of RSV to the respiratory tract, where it reaches its preferred cell type, apical ciliated epithelial cells [4]. Following entry into the host cell, RSV initiates a number of responses to develop and assemble RSV virions that then bud from the host surface and go on to infect other epithelial and immune cells [5,6]. Since viruses are non-living entities, a vital element of this process is the successful manipulation of the host cellular metabolism. In doing so, viruses are able to increase sources of energy and divert pools of free nucleotides and amino acids to achieve virion assembly [7]. This process during RSV infections has been only partially explored.

Key mediators of metabolic pathways under hypoxic and/or inflammatory conditions include the hypoxia-inducible factor (HIF) transcription factors (TFs) [8]. These TFs are heterodimers consisting of an alpha and beta subunit. In mammals, there are three paralogs of the HIF-α subunit (HIF-1α HIF-2α and HIF-3α) and two paralogs of HIF-β (ARNT/HIF-1β, ARNT2/HIF-2β) [9]. HIF-1 is considered to be the master regulator and is comprised of a constitutively expressed HIF-1β subunit that binds with a stabilized HIF-1α or HIF-2α to form the heterodimer complex. Once together, additional coactivators will interact with the HIF-1 complex to further regulate the transcription of target genes. Stabilization of the HIF-α subunit can occur in an oxygen-dependent (post-translational) and/or oxygen-independent (transcriptional) manner [10]. When oxygen is readily available, the HIF-α subunit is hydroxylated by prolyl-hydroxylases (PHDs), then polyubiquitinated by von Hippel–Lindau tumor suppressor protein (VHL) targeting it for proteasomal degradation [8,10]. This prevents the association of the two subunits and allows for normal cellular metabolism to continue. When hypoxic conditions arise, PHDs are inactivated, allowing for HIF-α accumulation and subsequent nuclear translocation. In the nucleus, HIF-α dimerizes with HIF-1β to form a complete HIF-1 complex that then recognizes hypoxia-response elements (HREs) to control target gene expression [8,10]. Alternatively, oxygen independent processes such as NFκB signaling following recognition of a pathogen by toll-like receptors (TLRs) or ligation of the T-cell receptor (TCR) during antigen presentation on T-lymphocytes can induce HIF gene transcription [8,11]. Genes targeted by HIF proteins encompass a variety of biological pathways such as metabolic reprogramming, proinflammatory cytokine secretion, redox homeostasis and apoptosis [11]. One of the better characterized is metabolic reprogramming. HIF-1 and 2 have been shown to upregulate all glycolytic enzymes to divert energy sources away from oxygen-demanding metabolic pathways towards glycolysis [8,11]. This would be preferable to a viral infection as glycolysis and the associated pentose phosphate pathway (PPP) can be used to increase the availability of needed materials such as nucleotides and amino acids for successful establishment of infection [7].

Previous studies with RSV using human bronchial epithelial cells (HBECs) and an experimental mouse model have demonstrated the stabilization of HIF-1α following infection [12,13], in part through RSV-induced nitric oxide-dependent pathways. Changes in core metabolic pathways in primary lung cells and the effects of HIF suppression during RSV infection have yet to be described. Here, we demonstrate a significant shift towards glycolysis and the PPP initiated during RSV infections in human small alveolar epithelial cells. Suppression of HIF-1α, but not HIF-2α, resulted in a reduction in hexokinase II enzyme, a key regulator of the glycolytic pathway, as well as in other HIF gene targets, such as pyruvate dehydrogease kinase 1 (PDK1) and vascular endothelial growth factor (VEGF). Inhibition of HIF-1α was also associated with a significant reduction in viral replication. Collectively, our study adds to the understanding of RSV-induced metabolic changes in primary airway epithelial cells and supports further investigation into the use of anti-HIF-1α therapeutics.

## 2. Materials and Methods

### 2.1. RSV Preparation, Cell Culture and Virus Infection

RSV Long strain was grown in HEp-2 cells (American Type Culture Collection, Manassas, VA, USA) and purified by polyethylene glycol precipitation, followed by centrifugation on 35–64% discontinuous sucrose gradients, as described elsewhere [14]. The titer of viral pools was determined by a methylcellulose plaque assay and ranged from 8–9 log10 plaque forming units (PFU)/mL. Virus pools were aliquoted, quick-frozen on dry ice-alcohol, and stored at −80 °C until needed.

Primary human small alveolar epithelial (SAE) cells (Lonza Walkersville, Inc., Walkersville, MD, USA) were derived from the terminal bronchioli of cadaveric donors. These cells were also utilized to generate immortalized SAE (iSAE), using human telomerase and cyclin dependent kinase-4 retrovirus constructs, as described in [15]. Both SAE and iSAE cells were cultured in growth medium containing 7.5 mg/mL bovine pituitary extract (BPE), 0.5 mg/mL hydrocortisone, 0.5 μg/mL human epidermal growth factor (hEGF), 0.5 mg/mL epinephrine, 10 mg/mL transferrin, 5 mg/mL insulin, 0.1 μg/mL retinoic acid, 0.5 μg/mL triiodothyronine, 50 mg/mL gentamicin, and 50 mg/mL bovine serum albumin (BSA). When cells were used for RSV infection, growth medium was replaced with basal medium lacking supplemental growth factors 6 h prior to infection and throughout the length of the experiments. Confluent cell monolayers were infected with RSV at multiplicity of infection (MOI) of 3 unless otherwise stated. For inhibition of HIF-1α or HIF-2α, iSAE cells were pretreated with PX-478 (10, 25, or 50 μM; Cayman Chemical Co., Ann Arbor, MI, USA) or PT-2385 (10 or 20 μM; Abcam, Cambridge, UK) for 1 h then infected with RSV in the presence of the compound. PX-478 suppresses HIF-1α in a variety of ways including inhibition of mRNA induction and protein translation [16]. PT-2385 suppresses HIF-2α by allosterically blocking the dimerization with ARNT/HIF-1β, thereby inhibiting the expression of many HIF-2α-dependent genes [17] Both inhibitors were prepared in dimethyl sulfoxide (DMSO), which was diluted at least 1:1000 at the final used concentrations, and equal amounts of diluent were added to untreated cells, as control. The total number of cells and cell viability, following inhibitor treatment, were measured by trypan blue exclusion. There was no significant change in cell viability with both compounds at all doses tested. To evaluate the effect of HIF-1α or HIF-2α inhibition on viral replication, HEp-2 and iSAE cells were infected at MOI of 0.1 and virus titer was measured by plaque assay in HEp-2 cells. Titers were calculated as PFU/mL.

### 2.2. Metabolomics Assay

Cell monolayers were infected with RSV for the indicated times. After removing the supernatant, cells were rinsed with 150 mM ammonium acetate in water buffer and liquid nitrogen was added directly onto the surface of the plates to rapidly freeze cells. Covered plates were transferred to a cooler with dry ice and stored at −80 °C until needed. Extraction and metabolomics analyses were done using gas chromatography-mass spectrometry (GC/MS) and liquid chromatography-mass spectrometry (LC/MS). Assays were performed by the University of Michigan Metabolomics Core.

### 2.3. Western Blots

Total cell and nuclear extracts were prepared as previously described [18,19]. Equal amount of protein (25 µg per sample) were then boiled in 2× Laemmli buffer and resolved on SDS-PAGE gels. Proteins were transferred onto a polyvinylidene difluoride membrane (Amersham, Piscataway, NJ, USA), and nonspecific binding sites were blocked by immersing the membrane in Tris-buffered saline (TBS) containing 5% skim milk powder, overnight at 4 °C. After a short wash in TBS-Tween (TBST), membranes were incubated with the primary antibody at the recommended dilution overnight at 4 °C. This was followed by incubation with horseradish peroxidase (HRP)-conjugated secondary antibody (Santa Cruz, CA, USA), diluted 1:5000 in TBS, for 1 h at room temperature. Finally, after washing three times with TBST, proteins were detected by using an enhanced chemiluminescence system (RPN 2016; Amersham, GE Healthcare, UK). Membranes were stripped and reprobed with anti-β-actin antibody (Sigma-Aldrich, St. Louis, MO, USA) for total cell lysate loading control or histone deacetylase-1 (HADC1; Cell Signaling, Danvers, MA, USA) for nuclear protein loading control. Densitometric analysis of band intensities was performed using UVP VisionWorksLS Image Acquisition and Analysis Software 8.0 RC 1.2 (UVP, Upland, CA, USA). The primary antibodies used for Western blot assays were HIF-1α rabbit mAB (Cell Signaling, Danvers, MA, USA), HIF-2α rabbit mAb (Novus Biologicals, Littleton, CO, USA), and HKII Rabbit mAb (Cell Signaling, Danvers, MA, USA).

### 2.4. Quantitative Real-Time PCR

RNA was extracted from iSAE cells using an Aurum Total RNA Mini Kit (BioRad, Hercules, CA, USA) according to the manufacturer instructions. RNA samples were quantified using a DS-11 Spectrophotometer (DeNovix Inc., Wilmington, DE, USA). Synthesis of cDNA was performed with 1 μg of total RNA in a 20 μL reaction using SuperScript III Reverse Transcriptase reagents according to the manufacturer’s instructions (Invitrogen, Carlsbad, CA, USA). Q-PCR amplification was done using 1 μL of cDNA in a total volume of 25 μL using a Taq DNA polymerase master mix. 18S RNA was used as a housekeeping gene for normalization. PCR assays were run in the BioRad CFX Connect Real-Time System. Duplicate CT values were analyzed in Microsoft Excel using the comparative CT (∆∆CT) method. The amount of target (2^−∆∆CT^) was obtained by normalizing to endogenous reference (18S) sample. Primer sequences are available upon request.

### 2.5. Statistical Analysis

The data were analyzed by one-way analysis of variance (ANOVA) followed by Tukey’s multiple comparisons test (GraphPad Prism 8; GraphPad Software Inc., San Diego, CA, USA). Results are expressed as mean ± SEM, and a *p* value < 0.05 was selected to indicate significance. Experiments were performed in triplicate when appropriate and repeated a minimum of two times.

## 3. Results

### 3.1. RSV Infection Induces a Shift in Core Metabolic Pathways in Human Primary Airway Epithelial Cells

**Figure 1:**
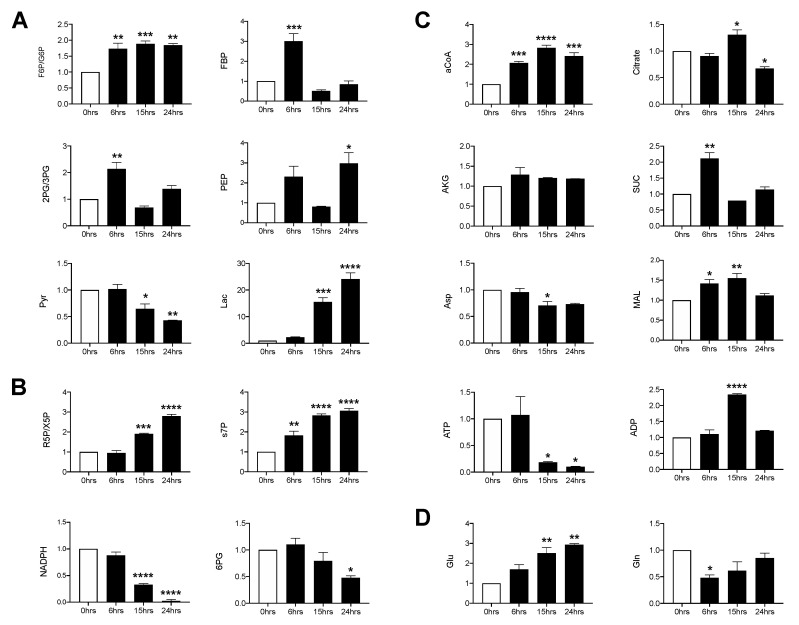
Metabolomic analysis of glycolysis/tricarboxylic acid (TCA)/pentose phosphate pathway (PPP). Small alveolar epithelial (SAE) cells were infected with respiratory syncytial virus (RSV) for 0, 6, 15, and 24 h at an MOI of 3. Cells were then harvested to measure metabolites found in the (**A**) glycolytic pathway, (**B**) pentose phosphate pathway, (**C**) TCA pathway, and the (**D**) glutamine pathway by GCMS/LCMS. F6P/G6P: fructose 6-phosphate/Glucose 6-phosphate; FBP: fructose 1,6, biphosphate; 2PG/3PG: 2-phosphoglycerate/3-phosphoglycerate; PEP: phosphoenolpyruvate; Pyr: pyruvate; Lac: lactate; R5P/X5P: ribulose-5-phosphate/xylulose-5-phosphate; s7P: sedoheptulose-7P; NADPH: nicotinamide adenine dinucleotide phosphate reduced; 6PG: 6-phospohogluconic acid; aCoA: acetyl-CoA; AKG: alpha-ketoglutarate; SUC: succinate; Asp: aspartate; MAL: malate; ATP: adenosine triphosphate; ADP: adenosine diphosphate; Glu: glutamate; Gln: glutamine. Data are presented as mean ± SEM. Significant results as compared to the uninfected control are marked with asterisks (* for *p* ≤ 0.05, ** *p* ≤ 0.01, *** *p* ≤ 0.001, **** *p* ≤ 0.0001).

To determine whether RSV infection was associated with changes in core metabolic pathways, SAE cells were infected for 6, 15 and 24 h and whole cell lysates were subjected to gas chromatography-mass spectrometry (GC/MS) and liquid chromatography-mass spectrometry (LC/MS) analysis of the glycolysis, tricarboxylic acid (TCA) and pentose phosphate pathway (PPP). In the glycolysis pathway, most metabolites were increased at the 6 h time post-infection (p.i.). They returned to levels similar to the uninfected control by 15 h p.i., with the exception of fructose-6-phosphate/glucose-6-phosphate (F6P/G6P) and phosphoenolpyruvate (PEP), which remained elevated at 24 h p.i. Pyruvate levels progressively decreased in the course of the infection, with a parallel increase in lactic acid levels (Figure 1A). Metabolites of the non-oxidative PPP, ribose-5-phosphate/xylulose 5-phosphate (R5P/X5P) and sedoheptulose 7-phosphate (s7P), increased in a time dependent manner while those of the oxidative branch, 6-phosphogluconate (6PG) and nicotinamide adenine dinucleotide phosphate hydrogen (NADPH), decreased throughout the infection period (Figure 1B). For the TCA cycle, acetyl coenzyme A (aCoA), succinate and malate were all initially increased (6 h p.i.) and subsequently decreased, with the exception of aCoA, which was maintained at a high level through the duration of the infection. Citrate increased at 15 h p.i., followed by a significant reduction at 24 h p.i. as compared to the uninfected control. Aspartate (Asp) decreased at 15 h p.i. and remained decreased, though not significantly, at 24 h p.i., while α-ketoglutarate (AKG) remained comparable to the uninfected control at all time points (Figure 1C). Additionally, glutamine was initially decreased and steadily recovered over time. Conversely, glutamate significantly increased over time as compared to the uninfected control (Figure 1D). Collectively, these data show that RSV infection significantly modulates glycolysis, the PPP and glutamine metabolism in primary SAE cells.

### 3.2. RSV Infection Stabilizes Expression of HIF-1α in SAE and Immortalized SAE Cells

**Figure 2:**
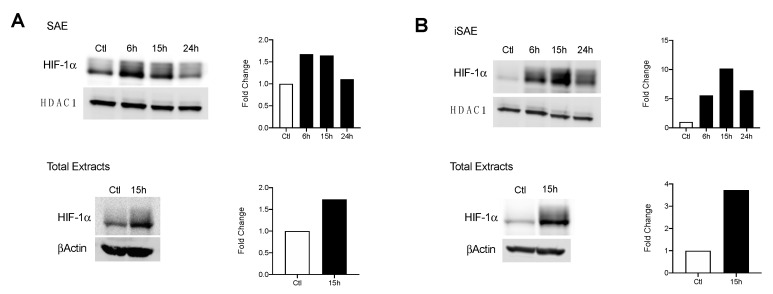
HIF-1α activation in response to RSV infection. Cells were infected with RSV and harvested at the indicated time points. Nuclear and total lysates of (**A**) SAE and (**B**) immortalized SAE (iSAE) cells were then subjected to Western blot analysis to determine HIF-1α levels. Western blot analyses shown here represent one independent experiment. All experiments were repeated at least twice. Densitometric analysis of band intensity, normalized to HDAC-1 for nuclear extracts and βActin for total lysates, is shown to the right of each blot.

Under hypoxic conditions, changes in HIF-1α expression allow for critical shifts in cellular metabolism. We initially assessed whether RSV infection was associated with enhanced HIF-1α nuclear levels. SAE cells were infected for 6, 15, and 24 h and harvested to prepare nuclear extracts. A 1.67-fold increase in HIF-1α was detected at 6 and 15 h p.i. with levels comparable to the uninfected control by 24 h p.i. (Figure 2A, top panel). A similar increase in HIF-1α expression was also detected in total cell lysates (Figure 2A, bottom panel). We next investigated HIF-1α levels in RSV-infected iSAE cells, which are telomerase-immortalized cells derived from primary SAE, able to divide for a prolonged number of passages and therefore more amenable to manipulations, such as gene deletions, than primary cells. HIF-1α expression in nuclear extracts increased 10-fold by 15 h p.i. before decreasing at 24 h p.i. (Figure 2B, top panel). Similar to SAE cells, increased HIF-1α expression was also detected in total cell lysates (Figure 2A, bottom panel). No change in HIF-1α gene expression was appreciated, as measured by RT-PCR (data not shown). Collectively, these data support stabilization of the HIF-1α protein in response to RSV infection in both primary and immortalized cells.

### 3.3. Suppression of HIF-1α Decreases Key Enzymes of the Glycolytic Pathway

**Figure 3:**
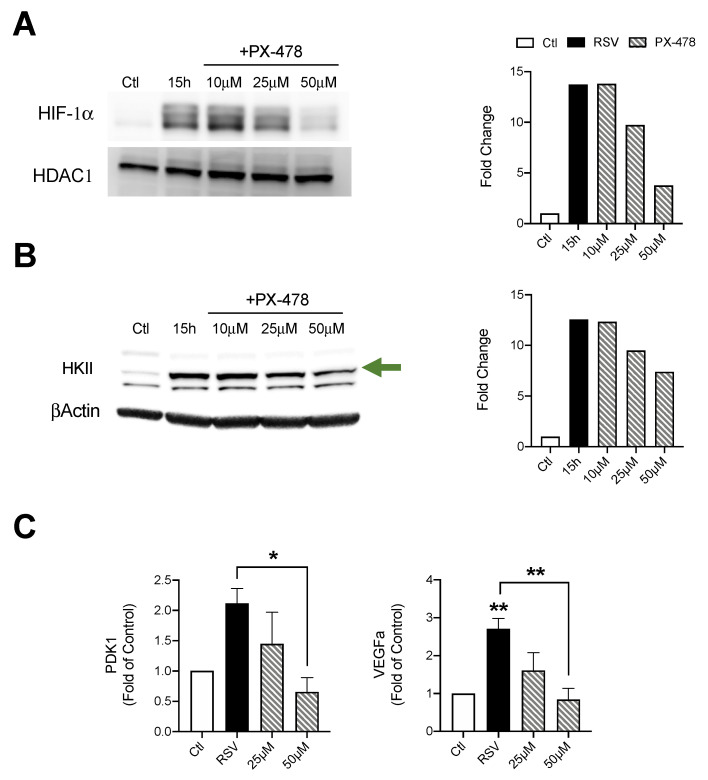
Effect of HIF-1α inhibition on metabolic enzyme expression. Cells were treated with PX-478 (10, 25, or 50 μM) for 1 h, followed by infection with RSV. Cells were harvested at 15 h p.i. to prepare nuclear extracts or total cells lysates. Expression of (**A**) HIF-1α and (**B**) HKII was determined by Western blot analysis. Densitometric analysis of band intensity, normalized to HDAC-1 for nuclear extracts and βActin for total lysates, is shown to the right. (**C**) RT-PCR was performed to measure gene expression of PDK1 and VEGFα. Data are presented as mean ± SEM. Western blot analyses shown here represent one independent experiment. All Western blot experiments were repeated at least twice. PCR analyses represent combined data from three independent experiments. Significant results as compared to the respective control are marked with asterisks, and additional comparisons between groups are indicated with brackets (* *p* ≤ 0.05, ** *p* ≤ 0.01). The green arrow indicates the target band at the expected 102kD molecular weight.

We next evaluated the effect of HIF-1α suppression during RSV infections. To achieve this, iSAE cells were treated with PX-478 (10, 25, or 50 μM) for 1 h, then infected with RSV. Cells were harvested at 15 h p.i. and expression of HIF-1α was determined by western blot analysis. The dose of 50 μM PX-478 had the greatest effect with a 10-fold reduction in HIF-1α expression as compared to the RSV infected control (Figure 3A). There was a significant increase in HKII protein levels in response to RSV infection, with a dose-dependent decrease following suppression of HIF-1α expression. The dose of 50 μM PX-478 resulted in the greatest reduction of 5.16-fold as compared to the RSV infected control (Figure 3B). Expression of pyruvate dehydrogease kinase 1 (PDK1), an enzyme that plays a key role in the regulation of metabolite flux through the TCA cycle, was also decreased in a dose-dependent manner, compared to the RSV infected control (Figure 3C). Similarly, RSV-induced expression of vascular endothelial growth factor A (VEGFa), a known gene associated with HIF-1α stabilization, was also significantly reduced by PX-478 treatment.

### 3.4. HIF-2α Expression Does Not Modulate Glycolytic Pathway in RSV Infection

**Figure 4:**
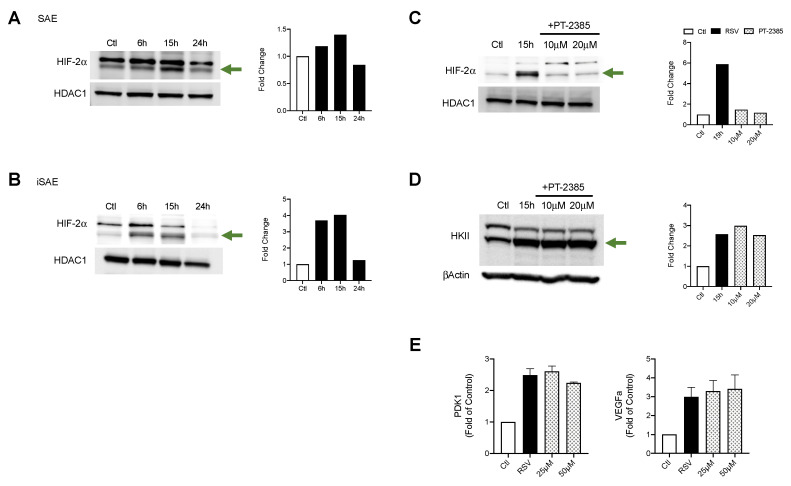
HIF-2α activation in RSV infection and role in metabolic gene expression. To assess the expression of HIF-2α, (**A**) SAE and (**B**) iSAE cells were infected with RSV followed by the collection of nuclear proteins at the indicated time points. iSAE cells were treated with PT-2385 (10 or 20 μM) for 1 h, followed by infection with RSV at an MOI of 3. Cells were harvested at 15 h p.i. and expression of (**C**) HIF-2α and (**D**) HKII was determined by Western blot analysis. Densitometric analysis of band intensity, normalized to HDAC-1 for nuclear lysates and βActin for total lysates, is shown to the right. (**E**) RT-PCR analysis was performed to measure gene expression of VEGFa and PDK1. Data are presented as mean ± SEM. Western blot analyses shown here represent one independent experiment. All Western blot experiments were repeated at least twice. PCR analyses represent combined data from three independent experiments. The green arrow indicates the target band at the expected 102 kD molecular weight.

HIF-2α activation is also known to affect metabolic activities as well as modulate expression of proinflammatory factors. To better understand the contributions of HIF-2α during RSV infections, we first assessed whether HIF-2α was activated in SAE and iSAE cells at 6, 15, and 24 h p.i. We found a 1.4-fold increase in HIF-2α nuclear levels in SAE cells beginning at 15 h p.i. that decreased at 24 h p.i. as compared to the uninfected control (Figure 4A). A similar pattern was observed in iSAE cells, with a 3.7-fold increase at 6 h p.i. and return to levels comparable to the uninfected control by 24 h p.i. (Figure 4B). Next, we determined the effect of HIF-2α inhibition on HKII expression during RSV infections. iSAE cells were treated with PT-2385 (10 or 20 μM) for 1 h, followed by infection with RSV. Cells were then harvested at 15 h p.i. and nuclear extracts were subjected to Western blot analysis. HIF-2α activation was effectively decreased with either dose of PT-2385 (Figure 4C), however, there was no significant reduction in HKII expression in RSV infected PT-2385-treated cells, compared to untreated (Figure 4D). Inhibition of HIF-2α also failed to modulate the expression of PDK1 or VEGFa (Figure 4E). Collectively, these data demonstrate that RSV infection can activate HIF-2α, but this HIF protein does not modulate the glycolytic pathway or expression of other canonical HIF-1α gene targets in the course of RSV infection.

### 3.5. Inhibition of HIF-1α Reduces Viral Replication

**Figure 5:**
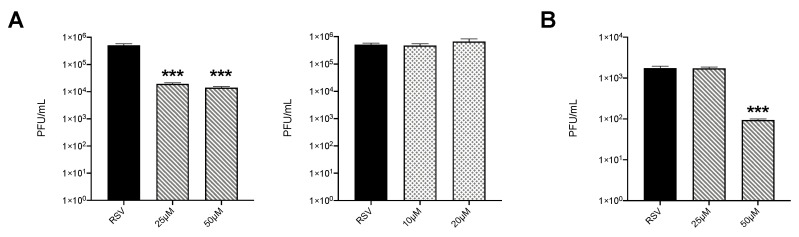
Effect of inhibition of HIF proteins on viral replication. (**A**) HEp-2 cells were infected with RSV at MOI of 0.1 in the presence or absence of PX-478 at 25 and 50 μM (left panel) or PT-2385 at 10 and 20 μM (right panel) and harvested at 36 h p.i. to determine viral titers by plaque assay. (**B**) iSAE cells were infected with RSV at MOI of 0.1 in the presence or absence of PX-478 at 25 and 50 μM and harvested at 36 h p.i. to determine viral titers by plaque assay. Viral titer analyses represent combined data from three independent experiments. Data are presented as mean ± SEM. Significant results as compared to the respective control are marked with asterisks, (*** *p* ≤ 0.001).

To determine whether inhibition of HIF proteins would affect RSV replication, HEp-2 cells were infected in the presence or absence of PX-478 or PT-2385. We found that both tested doses of PX-478 significantly reduced virus titers of almost two logs (Figure 5A left panel), while PT-2385 had no effect on viral replication (Figure 5A right panel). We then tested PX-478 in iSAE cells and found that the 50 μM dose was also able to reduce RSV replication by more than one log (Figure 5B).

## 4. Discussions

The ability of different viruses to reprogram host cell metabolism to promote viral replication has been an area of increasing investigations for its potential in developing novel therapeutic approaches. In this study, we investigated the metabolic changes induced by RSV infections followed by the effects of suppressing two key HIF subunits, HIF-1α and HIF-2α. To ensure there were no aberrant changes in metabolism as one might find in a cancer cell line, we assessed the metabolic changes in a model of human primary SAE cells, isolated from the terminal bronchioli of human donors [20]. Through LC/MS analysis, we found RSV to significantly modulate cellular metabolism towards the glycolytic and PPP pathways (Figure 1A,B). Of these metabolites, F6P/G6P appears to bottleneck, as FBP was only initially elevated before returning to levels comparable to the uninfected control. These metabolites are likely shunted to the PPP, supported by a time-dependent decrease in the oxidative branch and subsequent increase in the non-oxidative branch. In the non-oxidative branch, R5P/X5P and s7P were significantly increased while E4P (data not shown) was unaffected, suggesting that RSV prioritizes nucleotide production over amino acid production within the PPP. Additionally, we found decreasing amounts of pyruvate followed by significant increases in lactate production. This finding is indicative of increased aerobic glycolysis, also known as the Warburg effect. This phenomenon occurs when the majority of pyruvate is used for the production of lactate rather than being oxidized to aCoA for use in the TCA cycle, even in the presence of adequate oxygen concentrations [21]. As a result, glycolytic intermediates can be used for the synthesis of needed components for viral progeny assembly, such as nucleotides and amino acids.

Although the majority of TCA metabolites remained fairly similar in RSV-infected cells compared to uninfected controls (Figure 1C), aCoA was significantly elevated throughout the course of infection, while the production of ATP was significantly reduced. One possible explanation is that RSV upregulates the production of aCoA for palmytoilation of the RSV F protein during virion assembly, as previous studies have found this post-translational modification to be vital for intracellular replication of RSV [22,23]. To achieve this, aCoA would be reduced to citrate and then shuttled out of the mitochondria into the cytosol, where it would continue down the fatty acid biosynthesis pathway, stunting the efficiency of ATP production in the TCA cycle [24,25]. This theory is supported by a recent study examining the metabolic outcomes of RSV infections in human lung carcinoma (A549) cells [26]. Using LC/MS, they found palmitic acid to be one of the few lipids upregulated during the infection period, although our collective findings on the TCA cycle differ in that RSV infection in A459 cells seems to increase TCA activity and subsequent ATP production. This may be explained by inherent metabolic differences between primary and cancer cell lines [20].

Finally, the glutamine pathway generally serves as an alternative source of nitrogen and carbon for nucleotide and lipid production as well as to help drive ATP production by supplementing AKG in the TCA cycle [27,28]. We found infection with RSV to decrease glutamine production while increasing the concentration of glutamate (Figure 1D); however, AKG was unaffected throughout the course of infection (Figure 1C). This suggests that glutamine is not oxidized for the purpose of ATP production through the TCA cycle, but rather is likely used as an alternative source for other biosynthetic reactions.

We recently investigated core metabolic pathway changes in SAE cells following infection with human metapneumovirus (hMPV), another virus belonging to the *Pneumoviridae* family and an important cause of respiratory infection in children, as well as in the adult and elderly population [29]. Similar to RSV, hMPV was associated with an increase in the upstream glycolysis metabolites, a decrease in pyruvate levels and a significant increase in lactate. However, hMPV infection caused a more significant impairment of the TCA cycle and oxidative phosphorylation than RSV infection and a general decrease in the PPP pathway as well, indicating that similar viruses can elicit different metabolic responses in airway epithelial cells.

The activity of HIFs as a master regulator of cellular metabolism has been well characterized in a variety of cancer models [30]. Only over the last decade has the ability of viruses to manipulate this system for the benefit of viral growth been recognized [31]. For experiments assessing the suppression of HIF-1a and HIF-2a, we used a stable line of telomerase immortalized SAE cells. By immortalizing primary cells using the human telomerase reverse transcriptase (hTERT) technique, these cells are able to be passaged for an extended period of time without gaining oncogenic characteristics [15,32]. In our study, we found RSV infections in SAE and iSAE cells to stabilize both HIF-1α and HIF-2α following infection. This pattern of stabilization for HIF-1α following RSV infection was also found to occur in primary bronchial epithelial cells and A549 cells in varying degrees of expression [12,13]. To our knowledge, we are the first to demonstrate RSV-induced HIF-2α activation in human primary airway epithelial cells. Interestingly, only HIF-1α suppression had an effect on RSV-induced glycolytic activity, as measured by protein expression of HKII and gene expression of PDK1 (Figure 3B,C). Other studies examining the mechanistic functions of HIF-1α and HIF-2α have shown that either TF has regulatory activity in oxygen homeostasis, with HIF-1α playing a primary role in modulation of glycolysis [33]. When we investigated the effects of HIF suppression on RSV replication, we found similar outcomes, with only HIF-1a inhibition resulting in reductions in viral titer (Figure 5A,B).

Several viruses, often associated with chronic infections, such as hepatitis C virus, herpesviruses, human papillomavirus and immunodeficiency virus (HIV), have been shown to induce HIF-1α activation, leading to metabolic cellular reprogramming and promotion of virus replication [31,34,35]. Influenza virus infection was also able to induce HIF-1α activation in lung carcinoma and monocytic cell lines; however, a lack of lung epithelial cell expression of HIF-1α in a mouse model of influenza infection was associated with enhanced virus replication [36,37]. HIF-1α expression and stabilization in epithelial and immune cells plays an important role in promoting inflammatory responses, as well as the production of VEGF [8,33], both of which are known to contribute to the pathogenesis of RSV-induced lung disease, supporting further investigation into the use of selective HIF-1a inhibitors to modulate RSV disease, keeping in mind, however, that HIF proteins are also important for repair/healing processes following acute lung injury [38,39].
